# *catena*-Poly[[bis­[tris­(4-meth­oxy­phen­yl)phosphine-κ*P*]silver(I)]-μ-thio­cyanato-κ^2^*N*:*S*]

**DOI:** 10.1107/S2414314625008065

**Published:** 2025-09-23

**Authors:** Frederick P. Malan, Kariska Potgieter, Reinout Meijboom

**Affiliations:** a Department of Chemistry, University of Pretoria, Lynnwood Road, Hatfield, Pretoria, 0002, South Africa; b Department of Chemical Sciences, University of Johannesburg, PO Box 524, Auckland Park, 2006, Johannesburg, South Africa; Benemérita Universidad Autónoma de Puebla, México

**Keywords:** crystal structure, silver(I), tris­(4-meth­oxy­phen­yl)phosphine, thio­cyanato ligands, polymer

## Abstract

The coordination polymer, [Ag(NCS)(C_21_H_21_O_3_P)_2_]_*n*_ or {[Ag(P(4-OMePh)_3_)_2_]-μ-NCS}_*n*_, features μ-thio­cyanato ligands bridging between neighbouring silver(I) atoms, generating chains along the *b*-axis direction. Each Ag^I^ atom is four-coordinate, with two phospho­rus donors from two distinct tris­(*p*-meth­oxy­phen­yl)phosphine ligands, and two atoms from the thio­cyanato ligands in severely distorted tetra­hedral shape. The Ag—P [2.4331 (6) and 2.4625 (5) Å], Ag—N [2.339 (2) Å] and Ag—S [2.6760 (6) Å] bond lengths all appear within the expected ranges, with corresponding P—Ag—P and S—Ag—N angles of 129.610 (19) and 86.75 (6)°. In the crystal, the polymeric chains pack into layers parallel to (001) separated by the aryl groups of the phosphine ligands.

## Abstract 2

The synthesis and single crystal structure description of a silver(I) polymer featuring tris­(4-meth­oxy­phen­yl)phosphine and thio­cyanato ligands are described.

## Graphical abstract

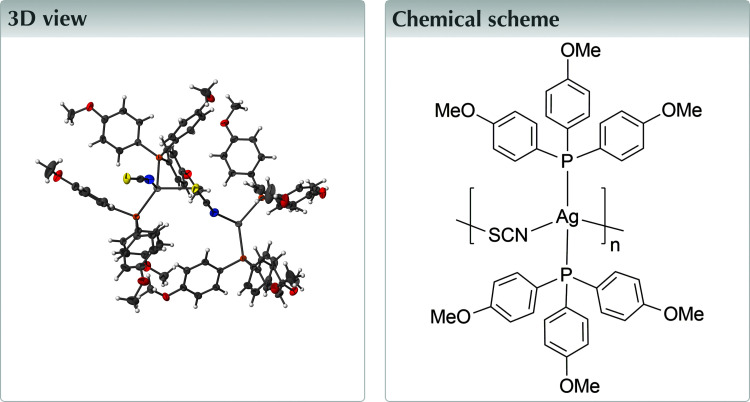

## Structure description

**Figure 1:**
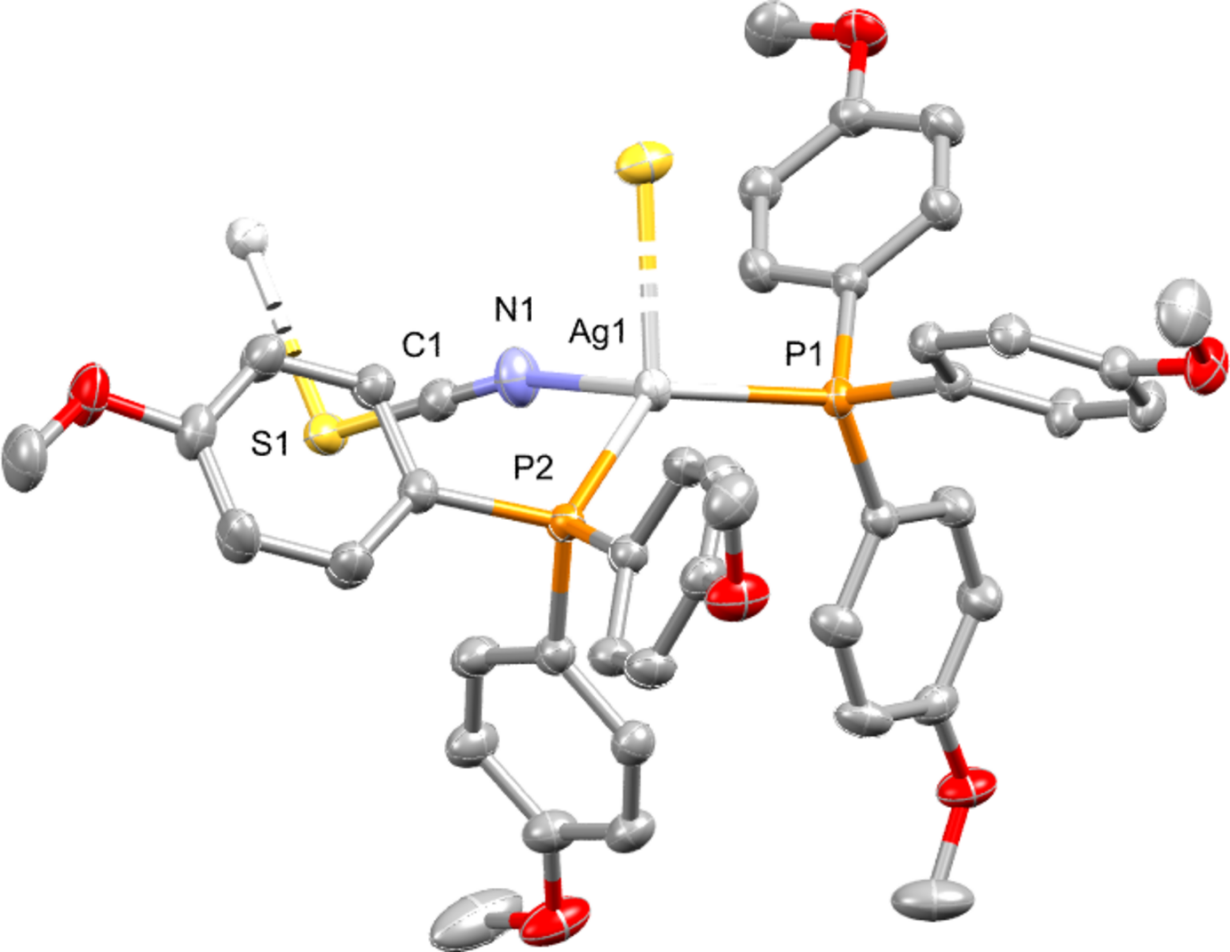
Mol­ecular structure of the title compound. Displacement ellipsoids are drawn at the 50% probability level. Hydrogen atoms are omitted for clarity.

**Figure 2:**
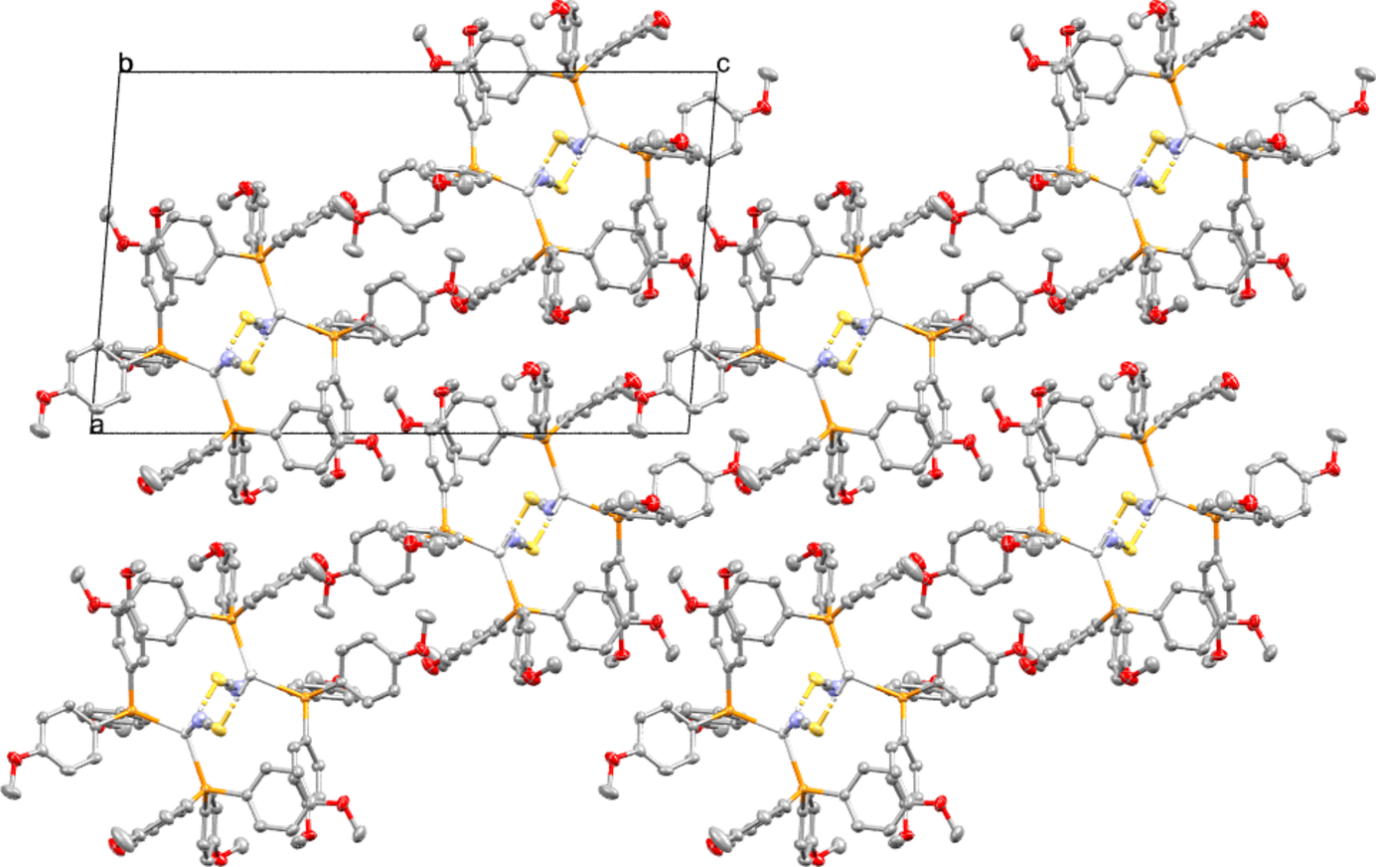
Packing diagram as viewed along the *b* axis.

Silver(I) phosphine complexes incorporating thio­cyanato ligands have been extensively investigated owing to their potential anti­microbial and anti­cancer activities, as well as their diverse structural motifs arising from the ambidentate nature of the SCN^−^ ligand (Engelbrecht *et al.*, 2018 ▸; Omondi & Meijboom, 2010 ▸; Naganagowda *et al.*, 2023 ▸). The thio­cyanate anions can coordinate *via* sulfur or nitro­gen atoms, or bridge between two central atoms, leading to polymeric motifs. The title complex, {[Ag(P(4-OMePh)_3_)_2_]-μ-NCS}_*n*_, crystallizes as a coordination polymer forming chains (Fig. 1 ▸). Each Ag^I^ atom is coordinated by two phospho­rus donors from two distinct tris­(4-meth­oxy­phen­yl)phosphine ligands, and two atoms from μ-bridging thio­cyanato ligands (one S and one N atom) producing a distorted tetra­hedral coordination environment. The Ag—P [2.4331 (6) and 2.4625 (5) Å], Ag—N [2.339 (2) Å] and Ag—S [2.6760 (6) Å] bond lengths are consistent with related structures. The S—Ag—N angle of the bridging ligand is 86.75 (6)°, which gives rise to the corrugated pattern that is observed of the resulting one-dimensional inorganic polymer. The packing diagram (Fig. 2 ▸) shows how polymer chains propagate along the *b-*axis direction and arrange into layers parallel to the (001) plane, with the bulky aryl substituents from the phosphine ligands forming hydro­phobic regions between the Ag—SCN-rich layers. Weak C—H⋯π contacts are observed between chains (Table 1 ▸), but no argentophilic or significant hydrogen-bonding inter­actions are evident.

## Synthesis and crystallization

A 1 mmol solution of silver thio­cyanate was prepared in 10 ml aceto­nitrile and carefully added to a solution of tris­(4-meth­oxy­phen­yl)phosphine (2 mmol) in 10 ml aceto­nitrile. The solution was stirred at 353 K for 12 h, removed and left to slowly cool to room temperature upon which colourless crystals formed.

## Refinement

Selected crystal data, data collection and structure refinement details are summarized in Table 2 ▸.

## Supplementary Material

Crystal structure: contains datablock(s) I. DOI: 10.1107/S2414314625008065/bh4101sup1.cif

Structure factors: contains datablock(s) I. DOI: 10.1107/S2414314625008065/bh4101Isup2.hkl

Supporting information file. DOI: 10.1107/S2414314625008065/bh4101Isup3.cdx

CCDC reference: 2486859

Additional supporting information:  crystallographic information; 3D view; checkCIF report

## Figures and Tables

**Table 1:** Hydrogen-bond geometry (Å, °)

*D*—H⋯*A*	*D*—H	H⋯*A*	*D*⋯*A*	*D*—H⋯*A*
C20—H20⋯S1^i^	0.95	2.85	3.765 (2)	162
C31—H31⋯O5^ii^	0.95	2.55	3.436 (3)	155
C7—H7⋯N1	0.95	2.62	3.457 (3)	148
C18—H18⋯O4^iii^	0.95	2.58	3.313 (3)	134
C34—H34⋯S1^i^	0.95	3.02	3.874 (3)	150
C8—H8*C*⋯O4^iv^	0.98	2.56	3.505 (3)	163
C36—H36*A*⋯O1^v^	0.98	2.64	3.588 (3)	164

Symmetry codes: (i) 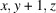; (ii) 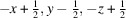; (iii) 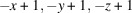; (iv) 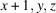; (v) 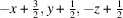.

**Table 2:** Experimental details

Crystal data	
Chemical formula	[Ag(NCS)(C_21_H_21_O_3_P)_2_]
*M*_r_	870.64
Crystal system, space group	Monoclinic, *P*2_1_/*n*
Temperature (K)	150
*a*, *b*, *c* (Å)	14.8676 (2), 11.1692 (2), 24.5206 (4)
β (°)	94.571 (1)
*V* (Å^3^)	4058.92 (11)
*Z*	4
Radiation type	Mo *K*α
μ (mm^−1^)	0.68
Crystal size (mm)	0.27 × 0.19 × 0.13

Data collection	
Diffractometer	XtaLAB Synergy R, DW system, HyPix
Absorption correction	Multi-scan (*CrysAlis PRO*; Rigaku OD, 2022 ▸)
*T*_min_, *T*_max_	0.644, 1.000
No. of measured, independent and observed [*I* > 2σ(*I*)] reflections	62976, 8935, 7505
*R*_int_	0.044
(sin θ/λ)_max_ (Å^−1^)	0.641

Refinement	
*R*[*F*^2^ > 2σ(*F*^2^)], *wR*(*F*^2^), *S*	0.031, 0.077, 1.06
No. of reflections	8935
No. of parameters	493
H-atom treatment	H-atom parameters constrained
Δρ_max_, Δρ_min_ (e Å^−3^)	0.49, −0.58

Computer programs: *CrysAlis PRO* (Rigaku OD, 2022 ▸), *SHELXT* (Sheldrick, 2015*a* ▸), *SHELXL* (Sheldrick, 2015*b* ▸) and *OLEX2* (Dolomanov *et al.*, 2009 ▸).

## full crystallographic data

### catena-Poly[[bis[tris(4-methoxyphenyl)phosphine-κP]silver(I)]-µ-thiocyanato-κ2N:S] Crystal data

**Table:**

[Ag(NCS)(C_21_H_21_O_3_P)_2_]	*F*(000) = 1792
*M**_r_* = 870.64	*D*_x_ = 1.425 Mg m^−^^3^
Monoclinic, *P*2_1_/*n*	Mo *K*α radiation, λ = 0.71073 Å
*a* = 14.8676 (2) Å	Cell parameters from 39976 reflections
*b* = 11.1692 (2) Å	θ = 2.5–31.1°
*c* = 24.5206 (4) Å	µ = 0.68 mm^−^^1^
β = 94.571 (1)°	*T* = 150 K
*V* = 4058.92 (11) Å^3^	Block, colourless
*Z* = 4	0.27 × 0.19 × 0.13 mm

### catena-Poly[[bis[tris(4-methoxyphenyl)phosphine-κP]silver(I)]-µ-thiocyanato-κ2N:S] Data collection

**Table:**

XtaLAB Synergy R, DW system, HyPix diffractometer	8935 independent reflections
Radiation source: Rotating-anode X-ray tube, Rigaku (Mo) X-ray Source	7505 reflections with *I* > 2σ(*I*)
Mirror monochromator	*R*_int_ = 0.044
Detector resolution: 10.0000 pixels mm^-1^	θ_max_ = 27.1°, θ_min_ = 2.4°
ω scans	*h* = −18→19
Absorption correction: multi-scan (CrysAlisPro; Rigaku OD, 2022)	*k* = −14→13
*T*_min_ = 0.644, *T*_max_ = 1.000	*l* = −31→30
62976 measured reflections	

### catena-Poly[[bis[tris(4-methoxyphenyl)phosphine-κP]silver(I)]-µ-thiocyanato-κ2N:S] Refinement

**Table:**

Refinement on *F*^2^	Primary atom site location: dual
Least-squares matrix: full	Secondary atom site location: difference Fourier map
*R*[*F*^2^ > 2σ(*F*^2^)] = 0.031	Hydrogen site location: inferred from neighbouring sites
*wR*(*F*^2^) = 0.077	H-atom parameters constrained
*S* = 1.06	*w* = 1/[σ^2^(*F*_o_^2^) + (0.0282*P*)^2^ + 4.1777*P*] where *P* = (*F*_o_^2^ + 2*F*_c_^2^)/3
8935 reflections	(Δ/σ)_max_ = 0.001
493 parameters	Δρ_max_ = 0.49 e Å^−^^3^
0 restraints	Δρ_min_ = −0.58 e Å^−^^3^

### catena-Poly[[bis[tris(4-methoxyphenyl)phosphine-κP]silver(I)]-µ-thiocyanato-κ2N:S] Fractional atomic coordinates and isotropic or equivalent isotropic displacement parameters (Å^2^)

**Table:**

	*x*	*y*	*z*	*U*_iso_*/*U*_eq_	
Ag1	0.66984 (2)	0.50887 (2)	0.30002 (2)	0.02483 (5)	
P1	0.73213 (4)	0.54588 (5)	0.39459 (2)	0.02116 (11)	
P2	0.51504 (4)	0.52475 (5)	0.26028 (2)	0.02381 (11)	
S1	0.68048 (4)	0.07281 (5)	0.24639 (3)	0.03463 (14)	
O2	0.59860 (12)	0.27681 (15)	0.58360 (7)	0.0384 (4)	
O1	1.11395 (11)	0.37447 (16)	0.41850 (7)	0.0371 (4)	
O6	0.47541 (13)	0.35429 (17)	0.02842 (7)	0.0443 (4)	
O3	0.69363 (13)	1.06316 (14)	0.44933 (7)	0.0385 (4)	
O4	0.35522 (14)	0.15703 (16)	0.40075 (8)	0.0498 (5)	
O5	0.32276 (13)	0.99377 (15)	0.22997 (8)	0.0440 (4)	
N1	0.70679 (15)	0.31120 (18)	0.27959 (9)	0.0398 (5)	
C9	0.68902 (14)	0.46807 (18)	0.45266 (9)	0.0229 (4)	
C16	0.72705 (14)	0.70339 (18)	0.41235 (8)	0.0227 (4)	
C21	0.70919 (14)	0.78583 (18)	0.37030 (9)	0.0250 (4)	
H21	0.7048	0.7584	0.3335	0.030*	
C2	0.85029 (14)	0.50274 (18)	0.40142 (8)	0.0221 (4)	
C10	0.60018 (15)	0.4293 (2)	0.45011 (10)	0.0301 (5)	
H10	0.5615	0.4462	0.4183	0.036*	
C20	0.69765 (15)	0.90707 (19)	0.38054 (9)	0.0270 (4)	
H20	0.6855	0.9617	0.3512	0.032*	
C30	0.45408 (15)	0.66641 (19)	0.25827 (9)	0.0262 (4)	
C31	0.35989 (15)	0.6769 (2)	0.25667 (9)	0.0297 (5)	
H31	0.3243	0.6077	0.2617	0.036*	
C1	0.69643 (15)	0.21315 (19)	0.26582 (9)	0.0261 (4)	
C35	0.50410 (15)	0.7704 (2)	0.25155 (9)	0.0300 (5)	
H35	0.5681	0.7654	0.2536	0.036*	
C37	0.49723 (15)	0.47051 (19)	0.19013 (9)	0.0268 (4)	
C17	0.73436 (17)	0.7458 (2)	0.46607 (9)	0.0305 (5)	
H17	0.7472	0.6914	0.4955	0.037*	
C27	0.37138 (16)	0.3585 (2)	0.37607 (10)	0.0324 (5)	
H27	0.3376	0.3796	0.4059	0.039*	
C28	0.40353 (15)	0.4474 (2)	0.34319 (9)	0.0300 (5)	
H28	0.3920	0.5291	0.3509	0.036*	
C14	0.74435 (16)	0.4418 (2)	0.49992 (9)	0.0303 (5)	
H14	0.8053	0.4680	0.5029	0.036*	
C6	0.95639 (15)	0.3426 (2)	0.38742 (9)	0.0287 (5)	
H6	0.9681	0.2643	0.3746	0.034*	
C7	0.86955 (15)	0.38898 (19)	0.38202 (9)	0.0269 (4)	
H7	0.8221	0.3422	0.3647	0.032*	
C23	0.45283 (14)	0.4175 (2)	0.29879 (9)	0.0262 (4)	
C3	0.92086 (15)	0.5715 (2)	0.42520 (9)	0.0285 (5)	
H3	0.9092	0.6496	0.4383	0.034*	
C5	1.02591 (14)	0.4116 (2)	0.41173 (9)	0.0274 (5)	
C19	0.70416 (15)	0.94696 (19)	0.43421 (9)	0.0281 (5)	
C12	0.62276 (16)	0.33994 (19)	0.53923 (9)	0.0294 (5)	
C42	0.57267 (16)	0.4433 (2)	0.16172 (9)	0.0309 (5)	
H42	0.6315	0.4516	0.1796	0.037*	
C40	0.47772 (17)	0.3922 (2)	0.08168 (9)	0.0330 (5)	
C32	0.31812 (16)	0.7869 (2)	0.24782 (10)	0.0331 (5)	
H32	0.2544	0.7930	0.2475	0.040*	
C24	0.46850 (16)	0.2966 (2)	0.28901 (10)	0.0339 (5)	
H24	0.5019	0.2749	0.2591	0.041*	
C11	0.56625 (15)	0.3662 (2)	0.49308 (11)	0.0340 (5)	
H11	0.5049	0.3414	0.4907	0.041*	
C26	0.38838 (16)	0.2386 (2)	0.36553 (10)	0.0345 (5)	
C41	0.56264 (17)	0.4047 (2)	0.10806 (10)	0.0352 (5)	
H41	0.6144	0.3866	0.0893	0.042*	
C13	0.71155 (16)	0.3784 (2)	0.54238 (9)	0.0330 (5)	
H13	0.7503	0.3609	0.5741	0.040*	
C4	1.00805 (16)	0.5267 (2)	0.42997 (10)	0.0326 (5)	
H4	1.0560	0.5748	0.4458	0.039*	
C25	0.43689 (17)	0.2075 (2)	0.32159 (11)	0.0373 (6)	
H25	0.4483	0.1258	0.3140	0.045*	
C18	0.72306 (17)	0.8662 (2)	0.47693 (9)	0.0333 (5)	
H18	0.7282	0.8939	0.5137	0.040*	
C34	0.46310 (16)	0.8807 (2)	0.24194 (10)	0.0336 (5)	
H34	0.4986	0.9501	0.2372	0.040*	
C39	0.40201 (17)	0.4172 (2)	0.10910 (10)	0.0393 (6)	
H39	0.3434	0.4082	0.0911	0.047*	
C8	1.13135 (18)	0.2540 (2)	0.40288 (11)	0.0408 (6)	
H8A	1.1127	0.2433	0.3639	0.061*	
H8B	1.0972	0.1989	0.4245	0.061*	
H8C	1.1960	0.2371	0.4094	0.061*	
C33	0.36983 (16)	0.8888 (2)	0.23934 (10)	0.0322 (5)	
C38	0.41245 (16)	0.4556 (2)	0.16334 (10)	0.0374 (6)	
H38	0.3605	0.4718	0.1822	0.045*	
C22	0.6868 (2)	1.1498 (2)	0.40634 (12)	0.0500 (7)	
H22A	0.6309	1.1365	0.3831	0.075*	
H22B	0.7386	1.1420	0.3843	0.075*	
H22C	0.6860	1.2304	0.4221	0.075*	
C36	0.3588 (2)	1.0762 (2)	0.19290 (11)	0.0435 (6)	
H36A	0.3666	1.0357	0.1581	0.065*	
H36B	0.4173	1.1057	0.2086	0.065*	
H36C	0.3172	1.1437	0.1865	0.065*	
C43	0.3909 (2)	0.3625 (3)	−0.00316 (11)	0.0502 (7)	
H43A	0.3990	0.3414	−0.0413	0.075*	
H43B	0.3679	0.4446	−0.0015	0.075*	
H43C	0.3478	0.3072	0.0116	0.075*	
C15	0.50676 (19)	0.2450 (3)	0.58537 (14)	0.0569 (8)	
H15A	0.4889	0.1913	0.5548	0.085*	
H15B	0.4694	0.3173	0.5826	0.085*	
H15C	0.4983	0.2042	0.6200	0.085*	
C29	0.3757 (3)	0.0334 (3)	0.39194 (18)	0.0846 (15)	
H29A	0.3500	0.0089	0.3556	0.127*	
H29B	0.3497	−0.0157	0.4199	0.127*	
H29C	0.4413	0.0224	0.3943	0.127*	

### catena-Poly[[bis[tris(4-methoxyphenyl)phosphine-κP]silver(I)]-µ-thiocyanato-κ2N:S] Atomic displacement parameters (Å^2^)

**Table:**

	*U*^11^	*U*^22^	*U*^33^	*U*^12^	*U*^13^	*U*^23^
Ag1	0.02521 (9)	0.02476 (9)	0.02393 (9)	−0.00037 (6)	−0.00173 (6)	−0.00318 (6)
P1	0.0235 (3)	0.0193 (2)	0.0204 (2)	−0.00024 (19)	0.0005 (2)	−0.00145 (19)
P2	0.0218 (3)	0.0293 (3)	0.0203 (3)	−0.0003 (2)	0.0015 (2)	−0.0013 (2)
S1	0.0364 (3)	0.0261 (3)	0.0433 (3)	−0.0071 (2)	0.0153 (3)	−0.0092 (2)
O2	0.0388 (10)	0.0378 (9)	0.0407 (10)	0.0002 (7)	0.0165 (8)	0.0114 (7)
O1	0.0223 (8)	0.0428 (10)	0.0456 (10)	0.0053 (7)	−0.0015 (7)	−0.0031 (8)
O6	0.0483 (11)	0.0557 (11)	0.0275 (9)	0.0094 (9)	−0.0060 (8)	−0.0127 (8)
O3	0.0557 (11)	0.0209 (8)	0.0396 (10)	0.0016 (7)	0.0082 (8)	−0.0056 (7)
O4	0.0600 (13)	0.0374 (10)	0.0566 (12)	0.0122 (9)	0.0330 (10)	0.0159 (9)
O5	0.0425 (10)	0.0306 (9)	0.0613 (12)	0.0091 (7)	0.0185 (9)	0.0065 (8)
N1	0.0485 (13)	0.0267 (10)	0.0434 (12)	0.0027 (9)	−0.0016 (10)	−0.0060 (9)
C9	0.0245 (10)	0.0190 (9)	0.0252 (10)	−0.0006 (8)	0.0028 (8)	−0.0017 (8)
C16	0.0248 (10)	0.0212 (10)	0.0223 (10)	−0.0011 (8)	0.0026 (8)	−0.0014 (8)
C21	0.0276 (11)	0.0248 (10)	0.0228 (10)	−0.0012 (8)	0.0034 (8)	−0.0025 (8)
C2	0.0216 (10)	0.0243 (10)	0.0204 (9)	−0.0011 (8)	0.0020 (8)	−0.0009 (8)
C10	0.0239 (11)	0.0279 (11)	0.0378 (13)	0.0025 (9)	−0.0016 (9)	0.0061 (9)
C20	0.0305 (12)	0.0221 (10)	0.0284 (11)	−0.0003 (8)	0.0023 (9)	0.0029 (8)
C30	0.0265 (11)	0.0286 (11)	0.0237 (11)	0.0003 (8)	0.0025 (8)	−0.0015 (8)
C31	0.0260 (11)	0.0316 (11)	0.0321 (12)	−0.0023 (9)	0.0064 (9)	0.0016 (9)
C1	0.0275 (11)	0.0285 (11)	0.0223 (10)	0.0025 (8)	0.0021 (8)	−0.0002 (8)
C35	0.0235 (11)	0.0346 (12)	0.0322 (12)	−0.0021 (9)	0.0035 (9)	−0.0043 (9)
C37	0.0281 (11)	0.0297 (11)	0.0222 (10)	−0.0002 (9)	−0.0003 (8)	0.0003 (8)
C17	0.0439 (14)	0.0263 (11)	0.0215 (11)	−0.0017 (9)	0.0039 (9)	0.0006 (8)
C27	0.0320 (12)	0.0381 (13)	0.0282 (12)	0.0034 (10)	0.0095 (10)	0.0017 (9)
C28	0.0303 (12)	0.0306 (11)	0.0292 (12)	0.0019 (9)	0.0034 (9)	−0.0016 (9)
C14	0.0278 (12)	0.0361 (12)	0.0266 (11)	−0.0074 (9)	−0.0007 (9)	0.0029 (9)
C6	0.0288 (12)	0.0268 (11)	0.0305 (12)	0.0040 (9)	0.0019 (9)	−0.0018 (9)
C7	0.0264 (11)	0.0242 (10)	0.0294 (11)	−0.0013 (8)	−0.0017 (9)	−0.0024 (8)
C23	0.0242 (11)	0.0312 (11)	0.0231 (10)	0.0008 (8)	0.0007 (8)	0.0001 (8)
C3	0.0277 (11)	0.0262 (11)	0.0311 (12)	−0.0017 (9)	−0.0005 (9)	−0.0043 (9)
C5	0.0226 (11)	0.0345 (12)	0.0252 (11)	0.0024 (9)	0.0015 (8)	0.0041 (9)
C19	0.0315 (12)	0.0216 (10)	0.0318 (12)	−0.0007 (8)	0.0054 (9)	−0.0034 (8)
C12	0.0344 (12)	0.0231 (10)	0.0323 (12)	0.0028 (9)	0.0124 (10)	0.0012 (9)
C42	0.0286 (12)	0.0377 (12)	0.0262 (11)	0.0004 (9)	0.0010 (9)	−0.0024 (9)
C40	0.0432 (14)	0.0307 (12)	0.0244 (11)	0.0059 (10)	−0.0012 (10)	−0.0031 (9)
C32	0.0247 (11)	0.0362 (12)	0.0391 (13)	0.0024 (9)	0.0071 (10)	0.0009 (10)
C24	0.0340 (13)	0.0346 (12)	0.0346 (13)	0.0004 (10)	0.0130 (10)	−0.0015 (10)
C11	0.0206 (11)	0.0321 (12)	0.0499 (15)	0.0005 (9)	0.0070 (10)	0.0079 (10)
C26	0.0326 (13)	0.0352 (12)	0.0369 (13)	0.0029 (10)	0.0101 (10)	0.0069 (10)
C41	0.0340 (13)	0.0441 (14)	0.0278 (12)	0.0060 (10)	0.0049 (10)	−0.0042 (10)
C13	0.0344 (13)	0.0390 (13)	0.0250 (11)	−0.0046 (10)	−0.0003 (9)	0.0036 (9)
C4	0.0246 (11)	0.0353 (12)	0.0375 (13)	−0.0048 (9)	−0.0003 (9)	−0.0051 (10)
C25	0.0408 (14)	0.0300 (12)	0.0430 (14)	0.0037 (10)	0.0150 (11)	−0.0002 (10)
C18	0.0497 (15)	0.0272 (11)	0.0236 (11)	−0.0040 (10)	0.0073 (10)	−0.0052 (9)
C34	0.0327 (13)	0.0284 (12)	0.0401 (13)	−0.0045 (9)	0.0061 (10)	−0.0049 (10)
C39	0.0302 (13)	0.0518 (15)	0.0340 (13)	0.0028 (11)	−0.0089 (10)	−0.0090 (11)
C8	0.0331 (13)	0.0445 (14)	0.0438 (15)	0.0135 (11)	−0.0027 (11)	−0.0023 (11)
C33	0.0334 (13)	0.0297 (11)	0.0341 (12)	0.0040 (9)	0.0071 (10)	−0.0014 (9)
C38	0.0262 (12)	0.0523 (15)	0.0336 (13)	0.0040 (11)	0.0016 (10)	−0.0089 (11)
C22	0.075 (2)	0.0218 (12)	0.0534 (17)	0.0066 (12)	0.0040 (15)	0.0028 (11)
C36	0.0533 (17)	0.0376 (14)	0.0404 (14)	0.0094 (12)	0.0093 (12)	0.0029 (11)
C43	0.0539 (18)	0.0620 (18)	0.0322 (14)	0.0033 (14)	−0.0113 (12)	−0.0105 (12)
C15	0.0382 (16)	0.0577 (18)	0.078 (2)	0.0017 (13)	0.0231 (15)	0.0277 (16)
C29	0.117 (3)	0.0375 (17)	0.110 (3)	0.0190 (19)	0.077 (3)	0.0246 (18)

### catena-Poly[[bis[tris(4-methoxyphenyl)phosphine-κP]silver(I)]-µ-thiocyanato-κ2N:S] Geometric parameters (Å, º)

**Table:**

Ag1—P1	2.4625 (5)	C14—H14	0.9500
Ag1—P2	2.4331 (6)	C14—C13	1.380 (3)
Ag1—S1^i^	2.6760 (6)	C6—H6	0.9500
Ag1—N1	2.339 (2)	C6—C7	1.388 (3)
P1—C9	1.827 (2)	C6—C5	1.386 (3)
P1—C16	1.815 (2)	C7—H7	0.9500
P1—C2	1.816 (2)	C23—C24	1.394 (3)
P2—C30	1.822 (2)	C3—H3	0.9500
P2—C37	1.823 (2)	C3—C4	1.386 (3)
P2—C23	1.823 (2)	C5—C4	1.393 (3)
S1—Ag1^ii^	2.6760 (6)	C19—C18	1.394 (3)
S1—C1	1.650 (2)	C12—C11	1.386 (3)
O2—C12	1.368 (3)	C12—C13	1.384 (3)
O2—C15	1.415 (3)	C42—H42	0.9500
O1—C5	1.371 (3)	C42—C41	1.382 (3)
O1—C8	1.428 (3)	C40—C41	1.379 (3)
O6—C40	1.370 (3)	C40—C39	1.385 (3)
O6—C43	1.425 (3)	C32—H32	0.9500
O3—C19	1.362 (3)	C32—C33	1.398 (3)
O3—C22	1.429 (3)	C24—H24	0.9500
O4—C26	1.374 (3)	C24—C25	1.382 (3)
O4—C29	1.434 (4)	C11—H11	0.9500
O5—C33	1.376 (3)	C26—C25	1.388 (3)
O5—C36	1.427 (3)	C41—H41	0.9500
N1—C1	1.153 (3)	C13—H13	0.9500
C9—C10	1.387 (3)	C4—H4	0.9500
C9—C14	1.398 (3)	C25—H25	0.9500
C16—C21	1.392 (3)	C18—H18	0.9500
C16—C17	1.396 (3)	C34—H34	0.9500
C21—H21	0.9500	C34—C33	1.386 (3)
C21—C20	1.390 (3)	C39—H39	0.9500
C2—C7	1.394 (3)	C39—C38	1.394 (3)
C2—C3	1.390 (3)	C8—H8A	0.9800
C10—H10	0.9500	C8—H8B	0.9800
C10—C11	1.395 (3)	C8—H8C	0.9800
C20—H20	0.9500	C38—H38	0.9500
C20—C19	1.386 (3)	C22—H22A	0.9800
C30—C31	1.403 (3)	C22—H22B	0.9800
C30—C35	1.396 (3)	C22—H22C	0.9800
C31—H31	0.9500	C36—H36A	0.9800
C31—C32	1.386 (3)	C36—H36B	0.9800
C35—H35	0.9500	C36—H36C	0.9800
C35—C34	1.386 (3)	C43—H43A	0.9800
C37—C42	1.400 (3)	C43—H43B	0.9800
C37—C38	1.384 (3)	C43—H43C	0.9800
C17—H17	0.9500	C15—H15A	0.9800
C17—C18	1.383 (3)	C15—H15B	0.9800
C27—H27	0.9500	C15—H15C	0.9800
C27—C28	1.388 (3)	C29—H29A	0.9800
C27—C26	1.390 (3)	C29—H29B	0.9800
C28—H28	0.9500	C29—H29C	0.9800
C28—C23	1.401 (3)		
			
P1—Ag1—S1^i^	95.31 (2)	C20—C19—C18	120.0 (2)
P2—Ag1—P1	129.610 (19)	O2—C12—C11	125.3 (2)
P2—Ag1—S1^i^	127.04 (2)	O2—C12—C13	115.2 (2)
N1—Ag1—P1	106.44 (6)	C13—C12—C11	119.6 (2)
N1—Ag1—P2	102.35 (6)	C37—C42—H42	119.6
N1—Ag1—S1^i^	86.75 (6)	C41—C42—C37	120.8 (2)
C9—P1—Ag1	121.67 (7)	C41—C42—H42	119.6
C16—P1—Ag1	111.65 (7)	O6—C40—C41	115.5 (2)
C16—P1—C9	104.48 (9)	O6—C40—C39	124.4 (2)
C16—P1—C2	107.05 (10)	C41—C40—C39	120.1 (2)
C2—P1—Ag1	109.31 (7)	C31—C32—H32	120.0
C2—P1—C9	101.46 (9)	C31—C32—C33	120.0 (2)
C30—P2—Ag1	121.79 (7)	C33—C32—H32	120.0
C30—P2—C37	103.21 (10)	C23—C24—H24	119.0
C30—P2—C23	108.24 (10)	C25—C24—C23	121.9 (2)
C37—P2—Ag1	114.53 (7)	C25—C24—H24	119.0
C23—P2—Ag1	104.33 (7)	C10—C11—H11	120.3
C23—P2—C37	103.22 (10)	C12—C11—C10	119.4 (2)
C1—S1—Ag1^ii^	105.72 (8)	C12—C11—H11	120.3
C12—O2—C15	118.0 (2)	O4—C26—C27	116.3 (2)
C5—O1—C8	116.38 (19)	O4—C26—C25	123.8 (2)
C40—O6—C43	116.9 (2)	C25—C26—C27	119.9 (2)
C19—O3—C22	116.61 (19)	C42—C41—H41	119.9
C26—O4—C29	117.0 (2)	C40—C41—C42	120.2 (2)
C33—O5—C36	116.38 (19)	C40—C41—H41	119.9
C1—N1—Ag1	158.6 (2)	C14—C13—C12	120.7 (2)
C10—C9—P1	120.35 (17)	C14—C13—H13	119.7
C10—C9—C14	117.9 (2)	C12—C13—H13	119.7
C14—C9—P1	121.73 (16)	C3—C4—C5	120.4 (2)
C21—C16—P1	118.19 (15)	C3—C4—H4	119.8
C21—C16—C17	118.06 (19)	C5—C4—H4	119.8
C17—C16—P1	123.61 (16)	C24—C25—C26	119.4 (2)
C16—C21—H21	119.0	C24—C25—H25	120.3
C20—C21—C16	122.0 (2)	C26—C25—H25	120.3
C20—C21—H21	119.0	C17—C18—C19	120.3 (2)
C7—C2—P1	115.70 (16)	C17—C18—H18	119.8
C3—C2—P1	125.74 (16)	C19—C18—H18	119.8
C3—C2—C7	118.5 (2)	C35—C34—H34	120.3
C9—C10—H10	119.2	C33—C34—C35	119.4 (2)
C9—C10—C11	121.6 (2)	C33—C34—H34	120.3
C11—C10—H10	119.2	C40—C39—H39	120.3
C21—C20—H20	120.5	C40—C39—C38	119.5 (2)
C19—C20—C21	119.0 (2)	C38—C39—H39	120.3
C19—C20—H20	120.5	O1—C8—H8A	109.5
C31—C30—P2	124.50 (17)	O1—C8—H8B	109.5
C35—C30—P2	117.20 (17)	O1—C8—H8C	109.5
C35—C30—C31	117.9 (2)	H8A—C8—H8B	109.5
C30—C31—H31	119.6	H8A—C8—H8C	109.5
C32—C31—C30	120.8 (2)	H8B—C8—H8C	109.5
C32—C31—H31	119.6	O5—C33—C32	116.1 (2)
N1—C1—S1	179.3 (2)	O5—C33—C34	123.8 (2)
C30—C35—H35	119.1	C34—C33—C32	120.0 (2)
C34—C35—C30	121.9 (2)	C37—C38—C39	121.2 (2)
C34—C35—H35	119.1	C37—C38—H38	119.4
C42—C37—P2	118.66 (17)	C39—C38—H38	119.4
C38—C37—P2	123.10 (18)	O3—C22—H22A	109.5
C38—C37—C42	118.2 (2)	O3—C22—H22B	109.5
C16—C17—H17	119.7	O3—C22—H22C	109.5
C18—C17—C16	120.7 (2)	H22A—C22—H22B	109.5
C18—C17—H17	119.7	H22A—C22—H22C	109.5
C28—C27—H27	119.8	H22B—C22—H22C	109.5
C28—C27—C26	120.3 (2)	O5—C36—H36A	109.5
C26—C27—H27	119.8	O5—C36—H36B	109.5
C27—C28—H28	119.8	O5—C36—H36C	109.5
C27—C28—C23	120.4 (2)	H36A—C36—H36B	109.5
C23—C28—H28	119.8	H36A—C36—H36C	109.5
C9—C14—H14	119.6	H36B—C36—H36C	109.5
C13—C14—C9	120.9 (2)	O6—C43—H43A	109.5
C13—C14—H14	119.6	O6—C43—H43B	109.5
C7—C6—H6	120.3	O6—C43—H43C	109.5
C5—C6—H6	120.3	H43A—C43—H43B	109.5
C5—C6—C7	119.3 (2)	H43A—C43—H43C	109.5
C2—C7—H7	119.2	H43B—C43—H43C	109.5
C6—C7—C2	121.5 (2)	O2—C15—H15A	109.5
C6—C7—H7	119.2	O2—C15—H15B	109.5
C28—C23—P2	124.36 (17)	O2—C15—H15C	109.5
C24—C23—P2	116.67 (17)	H15A—C15—H15B	109.5
C24—C23—C28	118.0 (2)	H15A—C15—H15C	109.5
C2—C3—H3	119.8	H15B—C15—H15C	109.5
C4—C3—C2	120.4 (2)	O4—C29—H29A	109.5
C4—C3—H3	119.8	O4—C29—H29B	109.5
O1—C5—C6	123.8 (2)	O4—C29—H29C	109.5
O1—C5—C4	116.4 (2)	H29A—C29—H29B	109.5
C6—C5—C4	119.8 (2)	H29A—C29—H29C	109.5
O3—C19—C20	124.3 (2)	H29B—C29—H29C	109.5
O3—C19—C18	115.7 (2)		
			
Ag1—P1—C9—C10	−28.5 (2)	C30—P2—C23—C28	−33.6 (2)
Ag1—P1—C9—C14	149.20 (16)	C30—P2—C23—C24	157.73 (18)
Ag1—P1—C16—C21	−13.67 (19)	C30—C31—C32—C33	1.2 (4)
Ag1—P1—C16—C17	162.10 (17)	C30—C35—C34—C33	0.5 (4)
Ag1—P1—C2—C7	−50.10 (17)	C31—C30—C35—C34	−1.9 (3)
Ag1—P1—C2—C3	131.66 (18)	C31—C32—C33—O5	179.0 (2)
Ag1—P2—C30—C31	−153.94 (16)	C31—C32—C33—C34	−2.6 (4)
Ag1—P2—C30—C35	33.7 (2)	C35—C30—C31—C32	1.0 (3)
Ag1—P2—C37—C42	−10.2 (2)	C35—C34—C33—O5	−179.9 (2)
Ag1—P2—C37—C38	170.20 (18)	C35—C34—C33—C32	1.7 (4)
Ag1—P2—C23—C28	97.42 (19)	C37—P2—C30—C31	75.7 (2)
Ag1—P2—C23—C24	−71.23 (18)	C37—P2—C30—C35	−96.58 (19)
P1—C9—C10—C11	177.81 (18)	C37—P2—C23—C28	−142.57 (19)
P1—C9—C14—C13	−177.14 (18)	C37—P2—C23—C24	48.8 (2)
P1—C16—C21—C20	175.26 (17)	C37—C42—C41—C40	−0.1 (4)
P1—C16—C17—C18	−175.03 (19)	C17—C16—C21—C20	−0.8 (3)
P1—C2—C7—C6	−176.52 (17)	C27—C28—C23—P2	−168.70 (18)
P1—C2—C3—C4	177.41 (18)	C27—C28—C23—C24	−0.2 (3)
P2—C30—C31—C32	−171.27 (18)	C27—C26—C25—C24	0.6 (4)
P2—C30—C35—C34	170.95 (18)	C28—C27—C26—O4	178.9 (2)
P2—C37—C42—C41	−178.45 (19)	C28—C27—C26—C25	−0.7 (4)
P2—C37—C38—C39	178.1 (2)	C28—C23—C24—C25	0.1 (4)
P2—C23—C24—C25	169.5 (2)	C14—C9—C10—C11	0.0 (3)
O2—C12—C11—C10	−178.6 (2)	C6—C5—C4—C3	1.6 (3)
O2—C12—C13—C14	179.3 (2)	C7—C2—C3—C4	−0.8 (3)
O1—C5—C4—C3	−179.5 (2)	C7—C6—C5—O1	−179.4 (2)
O6—C40—C41—C42	179.5 (2)	C7—C6—C5—C4	−0.5 (3)
O6—C40—C39—C38	−179.8 (2)	C23—P2—C30—C31	−33.2 (2)
O3—C19—C18—C17	179.5 (2)	C23—P2—C30—C35	154.46 (17)
O4—C26—C25—C24	−179.0 (2)	C23—P2—C37—C42	−123.00 (19)
C9—P1—C16—C21	−146.98 (17)	C23—P2—C37—C38	57.4 (2)
C9—P1—C16—C17	28.8 (2)	C23—C24—C25—C26	−0.3 (4)
C9—P1—C2—C7	79.59 (17)	C3—C2—C7—C6	1.9 (3)
C9—P1—C2—C3	−98.6 (2)	C5—C6—C7—C2	−1.2 (3)
C9—C10—C11—C12	−0.8 (4)	C42—C37—C38—C39	−1.5 (4)
C9—C14—C13—C12	−0.4 (4)	C40—C39—C38—C37	0.8 (4)
C16—P1—C9—C10	98.89 (19)	C11—C12—C13—C14	−0.4 (4)
C16—P1—C9—C14	−83.42 (19)	C26—C27—C28—C23	0.5 (4)
C16—P1—C2—C7	−171.19 (16)	C41—C40—C39—C38	0.3 (4)
C16—P1—C2—C3	10.6 (2)	C13—C12—C11—C10	1.0 (3)
C16—C21—C20—C19	0.0 (3)	C39—C40—C41—C42	−0.7 (4)
C16—C17—C18—C19	0.0 (4)	C8—O1—C5—C6	−4.8 (3)
C21—C16—C17—C18	0.7 (3)	C8—O1—C5—C4	176.3 (2)
C21—C20—C19—O3	−179.6 (2)	C38—C37—C42—C41	1.1 (4)
C21—C20—C19—C18	0.7 (3)	C22—O3—C19—C20	−8.1 (3)
C2—P1—C9—C10	−149.92 (18)	C22—O3—C19—C18	171.6 (2)
C2—P1—C9—C14	27.8 (2)	C36—O5—C33—C32	−145.4 (2)
C2—P1—C16—C21	105.92 (18)	C36—O5—C33—C34	36.2 (3)
C2—P1—C16—C17	−78.3 (2)	C43—O6—C40—C41	−168.2 (2)
C2—C3—C4—C5	−0.9 (4)	C43—O6—C40—C39	11.9 (4)
C10—C9—C14—C13	0.6 (3)	C15—O2—C12—C11	−6.4 (3)
C20—C19—C18—C17	−0.8 (4)	C15—O2—C12—C13	174.0 (2)
C30—P2—C37—C42	124.33 (19)	C29—O4—C26—C27	−177.3 (3)
C30—P2—C37—C38	−55.2 (2)	C29—O4—C26—C25	2.4 (4)

Symmetry codes: (i) −*x*+3/2, *y*+1/2, −*z*+1/2; (ii) −*x*+3/2, *y*−1/2, −*z*+1/2.

### catena-Poly[[bis[tris(4-methoxyphenyl)phosphine-κP]silver(I)]-µ-thiocyanato-κ2N:S] Hydrogen-bond geometry (Å, º)

**Table:**

*D*—H···*A*	*D*—H	H···*A*	*D*···*A*	*D*—H···*A*
C20—H20···S1^iii^	0.95	2.85	3.765 (2)	162
C31—H31···O5^iv^	0.95	2.55	3.436 (3)	155
C7—H7···N1	0.95	2.62	3.457 (3)	148
C18—H18···O4^v^	0.95	2.58	3.313 (3)	134
C34—H34···S1^iii^	0.95	3.02	3.874 (3)	150
C8—H8*C*···O4^vi^	0.98	2.56	3.505 (3)	163
C36—H36*A*···O1^i^	0.98	2.64	3.588 (3)	164

Symmetry codes: (i) −*x*+3/2, *y*+1/2, −*z*+1/2; (iii) *x*, *y*+1, *z*; (iv) −*x*+1/2, *y*−1/2, −*z*+1/2; (v) −*x*+1, −*y*+1, −*z*+1; (vi) *x*+1, *y*, *z*.
